# Cross-modular processing in a spiking neural network model

**DOI:** 10.1186/1471-2202-12-S1-P270

**Published:** 2011-07-18

**Authors:** Diogo PC Vieira, Antonio C Roque

**Affiliations:** 1 Departamento de Física, FFCLRP, Universidade de São Paulo, Ribeirão Preto, SP, 14040-901, Brazil

## 

According to the classical view about cortical organization, primary sensory areas are exclusively dedicated to a single sensory modality. Recent studies have challenged this view by showing that primary sensory cortices may undertake cross-modal processing whenever necessary [1,2]. Despite the growing interest in cross-modal cortical processing, the neural basis of this phenomenon remains largely unknown. How to understand cross-modal cortical processing given the anatomical structure of the cortex and the dynamic features of cortical neurons? It is known that cortical architecture is modular, so that neurons within a module are more densely connected among themselves than with neurons in other modules [3], and that the specificities of cortical connections depend on their range, with excitatory connections reaching longer distances than inhibitory connections [4]. It is also known that cortical neurons belong to different electrophysiological classes [5].

In this work we used a modular network of spiking neurons to study cross-modular processing in terms of average spike rate and latency. The network was made of 1,000 neurons modeled according to the Izhikevich formalism [6] to simulate three electrophysiological classes: fast spiking (FS), regular spiking (RS) and bursting (BS) neurons. Synaptic connections were modeled by *α*-functions. To generate the modular network, we started with a random network with a 4:1 ratio of excitatory to inhibitory neurons and sparse connectivity. The network was randomly divided into four modules of equal size. Each existing inter-modular inhibitory connection was deleted and corresponding intra-modular inhibitory connections were randomly created in turn. A similar rewiring was made for the inter-modular excitatory connections but with probability *p* of deleting an excitatory inter-modular connection. Furthermore, two modules (let us call them modules 1 and 2) were randomly selected and all connections between them were deleted. Three experimental protocols were performed: (a) the entire network was stimulated by random noise for 1200 msec; (b) similar to (a) but module 1 also received a fixed input pattern from t = 200 msec to t = 250 msec; and (c) the only input given to the network was the fixed pattern to module 1 only for the same time interval as in (b).

The raster plots of the four modules for protocol (a) show a similar frequency response for all of them but the spiking activity of module 2 cells is out of phase in comparison with activities in other modules. In the case of protocol (b), the presentation of the fixed input pattern to module 1 caused a slight and sustained increase in the average firing frequency of all modules. The start of this increase is delayed by 15 msec for modules 3 and 4, which are directly connected with module 1, and by 30 msec for module 2, which is only indirectly connected with module 1. These delays are not only due to synaptic delay (equal to 1 msec) but may also be due to intrinsic network properties. For protocol (c) the situation was similar to protocol (b) but with a lower average spike rate which decays toward zero after offset of the external stimulus. The simple network studied allow propagation of information between modules, with modules that do not receive direct external input responding stably (with delays) to an external and fixed input pattern presented to a single module. This suggests that more detailed analyses of modular networks may help understanding of cross-modal processing.

## References

[B1] Falchier A Clavagnier S Barone P Kennedy H Anatomical evidence of multimodal integration in primate striate cortex J Neurosci 2002 22 13 5749 5759 12097528 10.1523/JNEUROSCI.22-13-05749.2002 PMC6758216

[B2] Lakatos P Chen CM O’Connell MN Mills A Schroeder CE Neuronal oscillations and multisensory interaction in primary auditory cortex Neuron 2007 53 2 279 292 10.1016/j.neuron.2006.12.011 17224408 PMC3717319

[B3] Sporns O Chialvo D Kaiser M Hilgetag C Organization, development and function of complex brain networks Trends Cogn Sci 2004 8 9 418 425 10.1016/j.tics.2004.07.008 15350243

[B4] Bosking WH Zhang WH Schofield B Fitzpatrick D Orientation selectivity and the arrangement of horizontal connections in tree shrew striate cortex J. Neurosci 1997 17 6 2112 2127 9045738 10.1523/JNEUROSCI.17-06-02112.1997 PMC6793759

[B5] Contreras D Electrophysiological classes of neocortical neurons Neural Networks 2004 17 633 646 10.1016/j.neunet.2004.04.003 15288889

[B6] Izhikevich EM Simple model of spiking neurons IEEE Trans Neural Networks 2003 14 6 1569 1572 10.1109/TNN.2003.820440 18244602

